# NLRP3 Inflammasome Activation Mediates Coronary Artery Lesions in Kawasaki Disease by Inducing Endothelial Cells Pyroptosis and Glycocalyx Injury

**DOI:** 10.1002/iid3.70433

**Published:** 2026-04-13

**Authors:** Ronghao Zheng, Jing Yue, Qianjun Chen, Jing Xie, Lintao Wen, Nana Duan, Jianping Shang, Songbai Zhu, Li Huang, Yang Zou, Xiaoxiang Song, Xiaolin Wu, Qihua Feng

**Affiliations:** ^1^ Department of Rheumatology and Immunology Children's Hospital of Soochow University Suzhou Jiangsu Province China; ^2^ Department of Pediatric Nephrology, Rheumatology, and Immunology, Maternal and Child Health Hospital of Hubei Province, Tongji Medical College Huazhong University of Science and Technology Wuhan China; ^3^ Department of Respiratory Children's Hospital of Soochow University Suzhou Jiangsu China; ^4^ Emergency Department, Maternal and Child Health Hospital of Hubei Province, Tongji Medical College Huazhong University of Science and Technology Wuhan China; ^5^ Department of Pediatric Nephrology, Rheumatology, and Immunology, Maternal and Child Health Hospital of Hubei Province Hubei University of Medicine Shiyan Hubei China

**Keywords:** coronary artery lesions, glycocalyx injury, inflammatory, Kawasaki disease, NLRP3 inflammation, pyroptosis

## Abstract

**Introduction:**

To investigate the mechanism by which NOD‐like receptor heat protein domain‐associated protein 3 (NLRP3) activation mediates coronary endothelial dysfunction at different stages of Kawasaki disease (KD).

**Methods:**

Blood samples were collected from patients in the acute stage and convalescent stage of KD, as well as healthy/febrile controls. The mRNA expression of NLRP3 and Caspase‐1 were detected by qRT‐PCR. Serum levels of IL‐1β, syndecan‐1 (SDC‐1), hyaluronic acid (HA), metalloproteinase‐9 (MMP‐9), and metalloproteinase‐1 (TIMP‐1) were quantified via ELISA, and Spearman correlation analysis was conducted. In vitro, human coronary artery endothelial cells (HCAECs) were treated with KD patient serum combined with NLRP3 activator/inhibitor treatment. Cell viability (CCK‐8) and cell migration (Transwell) were assessed. The relative protein expression of pyroptosis‐related proteins (NLRP3, Caspase‐1, GSDMD‐N) were detected by Western blot. The concentrations of cytokine secretion (IL‐1β/IL‐18), matrix metabolism (MMP‐9/TIMP‐1), glycocalyx components (SDC‐1/HA) were evaluated by ELISA.

**Results:**

The levels of NLRP3 mRNA, Caspase‐1 mRNA, IL‐1β, MMP‐9, TIMP‐1, SDC‐1 and HA in patients with acute KD were significantly higher than those in the healthy/febrile control group (*p* < 0.01), and these indicators were positively correlated with NLRP3 activation (*p* < 0.01). KD serum significantly suppressed the proliferation/migration of HCAEC (*p* < 0.01), induced pyroptosis (upregulated NLRP3/Caspase‐1/GSDMD‐N), and increased IL‐1β/IL‐18 secretion with concomitant elevated levels of MMP‐9/TIMP‐1/SDC‐1/HA. NLRP3 inhibitors can reverse the above‐mentioned damaging effects (*p* < 0.01).

**Conclusions:**

NLRP3 inflammasome activation during the progression of KD can cause coronary endothelial injury by inducing inflammatory imbalance, endothelial cells pyroptosis, and glycocalyx injury. Targeting NLRP3 represents a promising therapeutic strategy.

## 1 Introduction

Kawasaki disease (KD) is a systemic vasculitis syndrome of unknown cause, which is more common in children under 5 years old [1]. Coronary artery lesions (CALs), as its main complication, are the core factor leading to the death of patients with KD [2]. Although intravenous immunoglobulin (IVIG) combined with high‐dose aspirin has become the standard therapy, some children still progress to CALs, significantly increasing the medical burden [3]. Studies have shown that endothelial permeability disruption plays a key role in the pathogenesis of KD vasculitis by mediating vascular leakage and inflammatory cell infiltration [4], but the specific mechanism by which it induces coronary endothelial dysfunction remains unclear.

The NLRP3 inflammasome has been confirmed to be involved in the pathological process of KD [5], and the expression of NLRP3 in the serum of patients is significantly upregulated [6, 7]. Its activation can promote the release of inflammatory factors such as IL‐1β and IL‐18 and induce pyroptosis [8]. Xu et al. [9] found, based on a mouse model, that human umbilical cord mesenchymal stem cells alleviate vascular endothelial injury by inhibiting the NLRP3 pathway‐related pyroptosis. Matrix metalloproteinases (MMPs) are a family of zinc‐dependent extracellular matrix (ECM) remodeling endopeptidases. Tissue inhibitors of MMPs (TIMPs) inhibit the proteolytic activity of MMPs, and their expression changes are important regulatory factors for ECM conversion, tissue remodeling, and cell behavior [10]. Studies have found that the expression of MMP‐9 and TIMP‐1 depends on the activation of IL‐1β [11]. The interaction between MMP‐9/TIMP‐1 and cytokines is a key feature of the occurrence and development of CALs and has become an important diagnostic marker [12]. The relationship and regulatory mechanism of NLRP3 and MMP‐9/TIMP‐1 in the occurrence of CALs in KD patients remain unclear.

The circulating glycocalyx component may be a new target for coronary artery protection in KD. Endothelial glycocalyx, as a gel layer covering vascular endothelium, is composed of proteoglycans and glycosaminoglycans (such as SDC‐1, HA), and directly regulates vascular permeability and inflammatory responses [13]. Clinical studies have confirmed that glucose calyx degradation markers such as SDC‐1 and HA in the serum of KD patients are significantly elevated, which is positively correlated with the degree of vascular endothelial injury [14, 15, 16]. Tang et al. found in the sepsis model that inhibiting the NLRP3 pathway could improve the integrity of the glycocalyx [17], suggesting a potential association between NLRP3 and glycocalyx injury. However, the relationship between the two in the progression of KD remains unclear.

Based on this, this study proposes a hypothesis: NLRP3 activation may jointly mediate KD coronary endothelial injury by inducing vascular inflammation by disrupting the MMP‐9/TIMP‐1 balance and damaging the endothelial barrier by inducing glycocalyx degradation. By analyzing the correlation between NLRP3 expression in clinical samples and inflammatory factors, CALs, and glycocalyx markers, and using the human coronary endothelial cell (HCAECs) model, combined with NLRP3 inhibitors, the effects of KD serum on cell biological behavior and glycocalyx were explored, aiming to clarify the dual mechanism of NLRP3‐mediated endothelial injury and provide a theoretical basis for targeted therapy.

## 2 Materials and Methods

### 2.1 Reagents and Instruments

Human coronary artery endothelial cells (HCAECs) and complete culture medium for human coronary artery endothelial cells (PY‐H045) were purchased from Shanghai Fuheng Biotechnology Co. Ltd. (China). 0.25% trypsin (T1350) was purchased from Beijing Solarbio (China). The CCK‐8 kit (BCCK0500), RIPA lysate (strong, containing inhibitors) (WB9201), and PVDF transfer membrane (BY09‐PW9230‐3) were purchased from Wuhan Bioswamp (China). FastPure Cell/Tissue Total RNA Isolation Kit V2 (RC112‐01) was purchased from Nanjing Vazyme Biotech Co. Ltd. (China). Hifair Ⅲ 1st Strand cDNA Synthesis SuperMix for qPCR (gDNA digester plus Hieff qPCR SYBR Green Master Mix (No Rox) (11141ES60) and Hifair Ⅲ 1st Strand cDNA Synthesis (11201ES08) were purchased from Shanghai Yeasen Biotechnology Co. Ltd. (China).

NLRP3 (JYM1921Hu), IL‐1β (JYM0083Hu), SDC‐1 (JYM2555Hu), HA (JYM0383Hu), MMP‐9 (JYM1104Hu), TIMP‐1 (JYM0316Hu) ELISA kits were purchased from Wuhan ColorfulGene Biotechnology Co. Ltd. (China). Antibody NLRP3 (PAB49005, 1:1000), Caspse‐1 (PAB36756, 1:1000), N‐ternimal gasdermin (GSDMD‐N, RMAB53302, 1:1000), Goat anti‐Rabbit IgG (BL003A, 1:20,000) and Goat anti‐mouse IgG (SAB43714, 1:20,000) were purchased from Wuhan Bioswamp (China). Antibody β‐actin (66009‐1‐lg, 1: 10000) was purchased from Proteintech.

The microplate reader (AMR‐100) was purchased from Hangzhou Aosheng Instrument Co. Ltd. (China). The inverted fluorescence microscope (DMIL LED) was purchased from Leica (Germany). The fluorescence quantitative PCR instrument (CFX‐Connect 96) was purchased from Bio‐Rad (American).

### 2.2 Clinical Sample Collection

After obtaining written informed consent, we enrolled the following participants: healthy controls (*n* = 10), febrile controls (*n* = 8), acute phase with CALs (*n* = 9), acute phase without CALs (*n* = 10), convalescent phase with CALs (*n* = 4), and convalescent phase without CALs (*n* = 10). Children with a median age of 24.0 months (range: 6.0–59.0 months) were diagnosed with KD according to the American Heart Association diagnostic criteria [18]. The onset of fever is regarded as the first day of the acute phase of KD. All patients with KD received a single dose of IVIG (2.0 g/kg) and 30–50 mg/kg/d of aspirin within 10 days of KD onset. In this study, blood samples for the acute phase were collected from 5 to 10 days after the onset of the disease, which occurs before IVIG treatment. The convalescent period was defined as after IVIG treatment and 72 h after the fever subsided, with CRP and ESR returning to normal. In this study, the blood samples of the convalescent period were collected 31–39 days after the onset of the disease. The coronary artery *Z*‐score was calculated based on echocardiography. A *Z*‐score of ≥ 2.0 indicated the presence of CAL. CAL that occurred during the acute phase or within 30 days of onset was defined as acute KD with CAL. CAL that persisted in the convalescent phase or newly emerged in the convalescent phase was defined as convalescent KD with CAL. In this study, no newly emerged CAL was found in the convalescent phase. Children of matched age, in good health and without any clinical symptoms of infection, were taken as the healthy control group. Children of similar age with only upper respiratory tract infection symptoms were selected as the febrile control group. Blood samples of all included children were collected from September 2024 to June 2025, and classified as above based on clinical data. This study was approved by the Medical Ethics Review Committee of Maternal and Child Health Hospital of Hubei Province (approval number: 2024‐017‐02).

Fasting venous blood samples were collected in the morning under standardized conditions: 3 mL of blood was collected in clot‐activator tubes, incubated at room temperature for 45 min, centrifuged at 2000*g* (4°C) for 15 min, extracted serum into EP tubes, and stored at −80°C for ELISA quantification of target proteins and subsequent cell experiments.

### 2.3 Cell Culture and Grouping

HCAECs were thawed, cultured in the complete culture medium of human coronary endothelial cells, and placed in a 37°C incubator with 5% CO_2_. The cell morphology was observed under an inverted microscope and passage cultured at a ratio of 1:2. HCAECs cells were divided into 96‐well plates, 3 × 10^3^ cells/well, 100 μL per well, and cultured overnight in a 5% CO_2_ incubator at 37°C to make the cells adhere to the wall. The serum of the normal control group, the serum of KD patients with CAL in the acute phase, the serum of KD patients without CAL in the acute phase and the serum of KD patients in convalescent phase (Note: only the serum of convalescent phase patients without CALs was used for intervention, because there were very few cases of convalescent phase KD patients with CAL in this study) were added respectively.

HCAECs cells were divided into 8 groups: Acute phase with CALs group (20% serum of acute phase with CALs patients were added), Acute phase without CALs group (20% serum of acute phase without CALs patients were added), convalescent phase group (20% serum of convalescent phase CALs patients were added), NLRP3 inhibitor group (20% serum of healthy control group and 10 μM NLRP3 inhibitor INF39 were added simultaneously), NLRP3 activator group (20% serum of healthy control serum and 10 μM NLRP3 activator1 were added simultaneously), Acute phase with CALs + NLRP3 inhibitor group (20% serum of acute phase with CALs patients and 10 μM NLRP3 inhibitor INF39 were added simultaneously), Acute phase without CALs + NLRP3 inhibitor group (20% serum of acute phase without CALs patients and 10 μM NLRP3 inhibitor INF39 were added simultaneously), and Healthy control group (20% serum of healthy control group were added).

### 2.4 Quantitative Reverse Transcription Polymerase Chain Reaction (qRT‐PCR)

After treatment, cells were lysed with Trizol to obtain total RNA. Strictly follow the method described in the kit, the cDNA product was obtained by reverse transcription and used as a template for amplification (20 μL system). PCR procedure: pre‐denaturation at 95°C for 3 min; subsequently, 40 cycles (95°C 5 s, 56°C 10 s, 72°C 25 s) were performed; finally, the melting curve was analyzed (starting at 65°C, heating up 0.5°C to 95°C every 5 s). PCR primers were synthesized by Wuhan Tianyi Huayu Gene Technology Co. Ltd. The primer sequences are as follows: Caspase‐1‐F: ATGGGCTCTGTTTTTATTGG; Caspase‐1‐R: TGTCCTGGGAAGAGGTAGAA; NLRP3‐F: TGAACAGCCACCTCACTT； NLRP3‐R: CAACCACAATCTCCGAAT; GAPDH‐F: GGGAAACTGTGGCGTGAT; GAPDH‐R: GAGTGGGTGTCGCTGTTGA.

### 2.5 CCK8 Detection

After the treatment of cells in each group was completed, 10 μL of CCK8 solution was added to each well, and the cells were further cultured for 1–4 h. The absorbance values of each well were measured at 450 nm using a microplate reader.

### 2.6 Enzyme‐Linked Immunosorbent Assay (ELISA) Detection

Firstly, the standard was diluted by gradient dilution, and then the standard hole, blank hole, and sample hole were set up on the microplate. The standard hole was added with 50 μL of different concentrations to dilute the standard, and the sample hole was added with 10 μL of protein sample and 40 μL of sample diluent after each group treatment. In addition to the blank holes, each hole was added with 50 μL enzyme‐labeled reagent, and incubated at 37°C for 30 min. After incubation, the samples were washed five times with 30 times diluted washing solution (each time filled with standing for 30 s after discarding and patting dry); add 50 μL reagent A and B to each well, and color at 37°C for 10 min in the dark. Finally, 50 μL termination solution was added to each hole to terminate the reaction, and the blank hole was zeroed within 15 min, and the OD value of each hole was measured at 450 nm wavelength. The experimental results were obtained by fitting the quadratic regression equation with the OD value of the standard as the abscissa and the concentration as the ordinate, and the OD value of the sample was substituted into the equation to calculate the actual concentration.

### 2.7 Transwell Test

The 24‐well plates and Transwell chambers were immersed in PBS for 5 min, and the cells were digested and washed with serum‐free medium. The cells were resuspended in a medium containing 1% FBS and adjusted to a density of 1 × 10^5^ cells/mL. Cell suspension of 0.5 mL after each treatment was added to the upper chamber, and 0.75 mL of medium containing 10% FBS was added to the corresponding hole in the lower chamber. The cells were cultured at 37°C for 24 h; subsequently, the medium was discarded, and PBS was gently washed once. Each well was added with 1 mL 4% formaldehyde and fixed at room temperature for 20 min. After the fixation solution was discarded, it was washed with PBS, stained with 1 mL 0.5% crystal violet for 30 min, and rinsed thoroughly with PBS three times. Finally, the non‐migrating cells in the upper chamber were wiped with a cotton swab, and the visual field was randomly selected under a 200‐fold microscope to count the migrating cells, and statistical analysis was performed.

### 2.8 Western‐Blot Detection

After the treatment of cells in each group, the cells were washed with pre‐cooled PBS, scraped, and collected with lysis solution, and pyrolyzed at 95°C. The lysate was centrifuged at 4°C to obtain the supernatant, and the protein concentration was quantified by the BCA method. After quantification, the sample was stored at −80°C. SDS‐PAGE gel was prepared. Before loading, 20 μg protein was boiled and denatured with the loading buffer. The supernatant was centrifuged and added to the gel hole. The electrophoresis was performed using a fast electrophoresis buffer at a constant pressure of 300 V for 20–30 min, and the membrane was transferred at 400 mA for 15–30 min. After the membrane was transferred, the membrane was immersed in a low background rapid blocking solution at room temperature for 10 min, and then incubated with primary antibody at room temperature for 1 h. After washing with PBST for three times, the membrane was incubated with HRP‐labeled secondary antibody (1: 10000) at room temperature for 1 h and washed. Finally, the ECL luminescent liquid A/B was mixed and covered on the surface of the film, and the bands were detected in the automatic chemiluminescence analyzer, and the gray value was analyzed by TANON GIS software.

### 2.9 Statistical Analysis

Data were expressed as mean ± standard deviation (mean ± SD). One‐way ANOVA (GraphPad Prism 9) was used for comparison between groups. Spearman's rank correlation was used for correlation analysis. The significance threshold was set to *p* < 0.05, and the significance level was marked with (*p < 0.05) and (**p < 0.01).

## 3 Results

### 3.1 The NLRP3 Inflammasome Is Activated and Positively Correlated With Endothelial Cells Pyroptosis and Glycocalyx Injury

**Figure 1:**
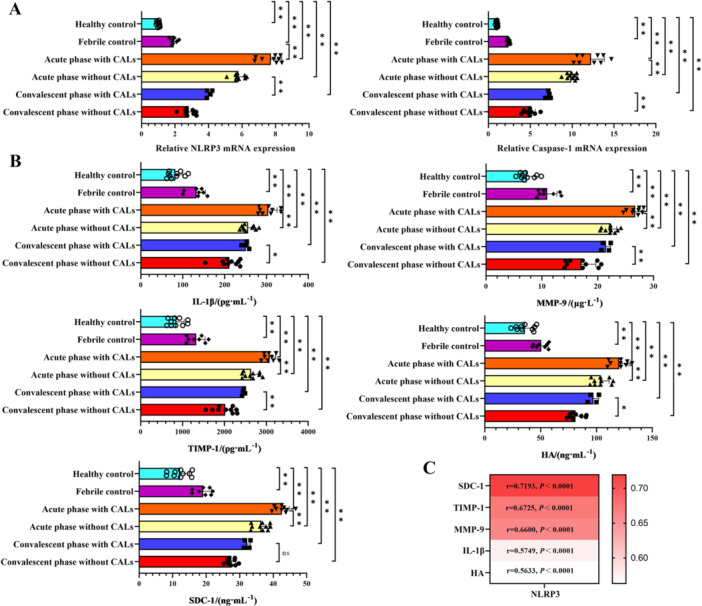
The NLRP3 inflammasome is activated and closely related to endothelial inflammation and glycocalyx injury. (A) The mRNA expression of NLRP3 and Caspase‐1 in the blood of patients detected by qRT‐PCR. (B) The concentrations of IL‐1β, MMP‐9, TIMP‐1, SDC‐1, and HA in the serum of patients by ELISA. (C) Spearman correlation analysis. **p < 0.01, *p < 0.05.

The activation status of the NLRP3 inflammasome in KD patients were evaluated in clinical samples. As shown in Figure 1A, compared with the healthy control group, the mRNA expression levels of NLRP3 and Caspase‐1 in the febrile control group and KD patients in the acute/convalescent stage were significantly up‐regulated (*p* < 0.01). The increases were more significant in patients with CALs than in those without CALs. Compared with KD patients in the acute stage, the mRNA expression levels of NLRP3 and Caspase‐1 in KD patients in the convalescent stage were decreased, but still higher than the healthy control group and the febrile group.

In parallel, the inflammatory state of the coronary artery and endothelial barrier function in KD patients were evaluated. As shown in Figure 1B, the serum concentrations of IL‐1β, MMP‐9, TIMP‐1, SDC‐1, and HA were markedly higher in KD patients both in the acute/convalescent stage than in healthy or febrile controls (*p *< 0.01). Compared with patients without CALs, the increases were more significant in patients with CALs. Levels of these indicators peaked in acute‐stage patients with CALs, decreased during convalescence, yet persisted above the healthy or febrile control values. As shown in Figure 1C, Spearman correlation analysis further revealed a strong positive association between the mRNA expression level of NLRP3 and IL‐1β, MMP‐9, TIMP‐1, SDC‐1, and HA (*p* < 0.0001) in acute stage KD patients.

### 3.2 KD Patient Serum Inhibits Endothelial Cell Function Through NLRP3‐Dependent Pathway

**Figure 2:**
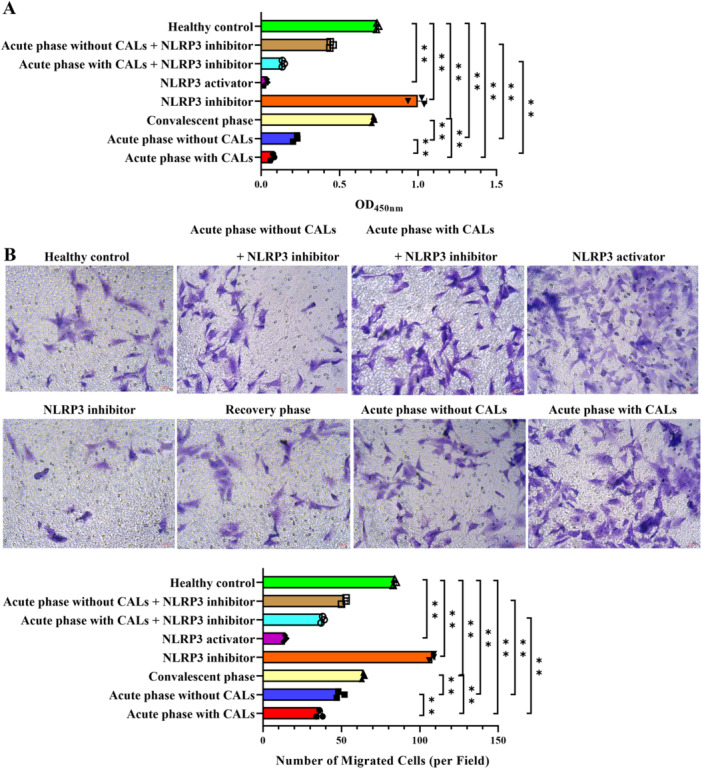
NLRP3 inflammasome activation inhibits the biological behavior of endothelial cells. (A) The cell proliferation detected by CCK8. (B) The migration ability detected by the transwell test (×200). **p < 0.01.

The effect of KD serum on the biological behavior of endothelial cells was investigated. As shown in Figure 2A,B, compared with the healthy control group, serum from KD patients in the acute phase (with/without CALs) and convalescent phase significantly inhibited cell proliferation and migration (*p* < 0.01). The suppressive effect was more severe with serum from acute‐stage CALs patients and was partly attenuated in convalescent‐stage samples. Importantly, the impairment was NLRP3‐dependent: an NLRP3 activator further reduced proliferation and migration, whereas an NLRP3 inhibitor significantly restored both cellular activities.

### 3.3 NLRP3 Inflammation Promotes KD Serum‐Induced Pyroptosis of Endothelial Cells

**Figure 3:**
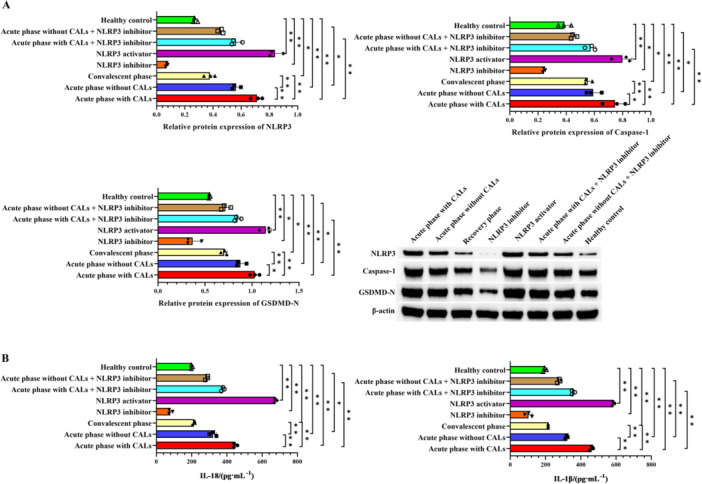
NLRP3 inflammasome activation promotes pyroptosis of endothelial cells. (A) The relative expression of NLRP3, Caspase‐1, and GSDMD‐N analyzed by Western blot. (B) The concentrations of IL‐18 and IL‐1β detected by ELISA. **p < 0.01, *p < 0.05.

The expression of pyroptosis‐related proteins was analyzed by Western blot. As shown in Figure 3A,B, relative to the healthy control group, KD serum significantly up‐regulated the expression of NLRP3, Caspase‐1, GSDMD‐N, IL‐18, and IL‐1β (*p* < 0.05). This increase was significantly more pronounced in acute‐stage KD patients with CALs than in those without CALs. Consistent with a central role for NLRP3, pharmacological activation of NLRP3 enhanced, while its inhibition attenuated, the expression of these pyroptosis‐related markers.

### 3.4 NLRP3 Activation Promotes the Release of Inflammatory Factors and Glycocalyx Damage in Endothelial Cells

**Figure 4:**
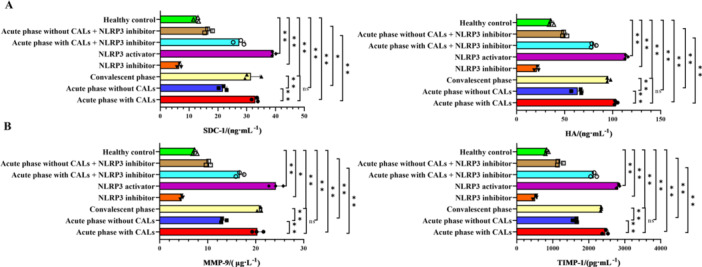
NLRP3 inflammasome activation induces the release of inflammatory factors and glycocalyx injury in endothelial cells. (A) The levels of SDC‐1 and HA detected by ELISA. (B) The levels of MMP‐9 and TIMP‐1 detected by ELISA. **p < 0.01, *p < 0.05.

The content of glycocalyx components and matrix metabolism‐related proteins was detected by ELISA, and the results are shown in Figure 4A,B. KD serum significantly increased the release of glycocalyx components (SDC‐1 and HA) and elevated levels of the matrix regulators MMP‐9 and TIMP‐1 in cell supernatants compared with the healthy control group (*p* < 0.05). Changes followed a consistent severity gradient: acute CALs >acute without CALs > convalescent. These effects were modulated by NLRP3 signaling, as they were amplified by an NLRP3 activator and suppressed by an NLRP3 inhibitor, indicating that NLRP3 activation drives glycocalyx injury and matrix imbalance in this setting.

## 4 Discussion

This study systematically elucidates the key role of NLRP3 inflammasome in the development of CALs in KD patients with coronary artery disease. The results showed that coronary artery inflammatory injury was present in different stages of KD patients, which was characterized by increased pro‐inflammatory cytokine IL‐1β, up‐regulation of MMP‐9/TIMP‐1 expression, and increased levels of endothelial glycocalyx degradation markers (SDC‐1/HA). It is worth noting that these pathological changes were significantly positively correlated with the activation of NLRP3 inflammasome. In vitro experiments further showed that serum intervention of KD patients could significantly inhibit the proliferation and migration of HCAECs and promote pyroptosis. NLRP3 inhibitors can partially reverse such damages. These results suggest that NLRP3 inflammasome activation‐driven vascular inflammation and endothelial glycocalyx injury are the core pathological mechanisms of KD progression to CALs.

NLRP3 inflammasome and its downstream IL‐1 signaling pathway have been shown to be closely related to KD vasculitis [19]. As a typical inflammatory disease, the occurrence and development of KD vasculitis depend on the activation of the innate immune system, which leads to the infiltration of immune effector cells and the destruction of perivascular tissues [20]. Clinical observations have found that high levels of pro‐inflammatory cytokines (such as IL‐1, IL‐18, and tumor necrosis factor [TNF]) in the circulation are significantly associated with the risk of CALs [21]. After activation of NLRP3 inflammasome, caspase‐1 cleavage promotes the secretion of active IL‐1β/IL‐18 and regulates the innate immune response, which is associated with a variety of inflammatory diseases, including KD [5, 22]. The coronary artery is the most important target organ of KD, and the main manifestations of CALs are coronary artery dilatation and aneurysm. Li et al. found that serum NLRP3 levels were elevated in KD patients and could be used as a potential marker for coronary artery aneurysm [23, 24]. The results of this study further confirmed this finding, especially in the acute phase KD patients with CALs, the levels of NLRP3mRNA and IL‐1β in peripheral blood were significantly higher than those in healthy controls and in the acute phase KD patients without CALs.

In terms of matrix metabolism, the MMP/TIMP system is associated with the clinical outcome of coronary artery disease. The interaction between MMP‐9/TIMP‐1 and inflammatory cytokines is a key molecular feature of the occurrence and development of CALs, and has become an important diagnostic marker [25]. It is worth noting that the expression of MMPs/TIMPs in serum of children with KD‐related CALs showed dynamic changes: although there was no significant difference between the CALs persistent group and the regression group during the fever period (*p* > 0.05), after treatment, the TIMP‐1 level in the CALs persistent group was significantly decreased (*p* < 0.05), suggesting that the insufficient expression of TIMP‐1 may be related to the persistence of CALs [26]. This study found that the levels of MMP‐9 and TIMP‐1 in the peripheral blood of acute phase KD patients (especially with CALs) were significantly increased, which was consistent with the report of Liu et al. [27]. More importantly, our correlation analysis revealed for the first time that the imbalance of MMP‐9/TIMP‐1 system was significantly correlated with the activation level of the NLRP3/IL‐1β pathway, suggesting that the NLRP3 inflammasome may disrupt MMP‐9/TIMP‐1 by promoting the release of inflammatory factors.

In recent years, the role of endothelial glycocalyx in the pathogenesis of KD has attracted increasing attention, and KD vasculitis may induce long‐term glycocalyx damage [14]. This study found that the serum glycocalyx components (SDC‐1 and HA) in KD patients were significantly increased in all disease stages, and the increase in patients with CALs in the acute phase was the most significant. In particular, the receiver operating characteristic (ROC) curve analysis showed that the combined detection of SDC‐1 and HA had a higher predictive value for CALs (AUC = 0.743), suggesting that these indicators may be potential biomarkers for CALs [28]. This finding is consistent with the conclusion of Luo et al. [14]. Although the association between NLRP3 and glycocalyx is limited, Feng et al. found that SDC1 may act as a bridge between hypoxia‐inducible factor‐1α (HIF‐1α) and NLRP3 inflammasome under hypoxic conditions based on the hemorrhagic shock model [29]. Tang et al. [17] found that inhibition of NLRP3 can significantly restore the injury of endodermal glycocalyx in rats with septic shock. This study confirmed for the first time in the KD model that patient serum can activate NLRP3 inflammasome in HCAECs, leading to up‐regulation of SDC‐1 and HA expression, while inhibiting cell proliferation and migration and promoting pyroptosis. NLRP3 inhibitor treatment significantly reduced these glycocalyx damage effects. These results clearly suggest that NLRP3 activation may be involved in the occurrence of CALs by promoting the release of inflammatory factors and leading to endothelial glycocalyx damage.

It should be pointed out that there are some limitations in this study. Firstly, there is a lack of direct observation of the ultrastructure of glycocalyx in coronary endothelial cells by transmission electron microscopy. Secondly, no KD animal model has been established to systematically evaluate the dynamic changes of coronary artery injury, inflammatory infiltration, and endothelial barrier integrity. These works will be focused on in the follow‐up study.

## 5 Conclusion

The abnormal activation of the NLRP3 inflammasome in the acute phase of Kawasaki disease (KD) leads to the massive release of inflammatory factors such as interleukin IL‐1β and IL‐18 by promoting Caspase‐1 cleavage and GSDMD‐mediated pyroptosis of endothelial cells, It also causes glycalyx degradation (increase of SDC‐1 and HA) and matrix metabolic disorders (imbalance of MMP‐9/TIMP‐1), and further induces coronary endothelial cell dysfunction, manifested as a significant decrease in proliferation and migration ability. These changes are particularly significant in the acute stage of the disease and are closely and positively correlated with NLRP3 activity. The application of NLRP3 inhibitors can effectively reverse the above‐mentioned pathological effects. Therefore, the activation of the NLRP3 inflammasome is one of the key mechanisms of coronary endothelial injury in Kawasaki disease. Targeted inhibition of the NLRP3 signaling pathway may provide a new strategy for the treatment of this.

## Author Contributions

**Ronghao Zheng:** conceptualization, investigation, data curation, methodology, formal analysis, writing – original draft, writing – review and editing, validation, funding acquisition, project administration. **Jing Yue:** conceptualization, methodology, data curation, investigation, formal analysis, writing – original draft, validation. **Qianjun Chen:** data curation, investigation, formal analysis, writing – original draft. **Jing Xie:** data curation, investigation, formal analysis, writing – original draft. **Lintao Wen:** data curation, formal analysis. **Nana Duan:** data curation, investigation. **Jianping Shang:** data curation. **Songbai Zhu:** data curation, investigation, formal analysis. **Li Huang:** data curation, investigation. **Yang Zou:** data curation, investigation. **Xiaoxiang Song:** writing – review and editing, resources, methodology, conceptualization, visualization. **Xiaolin Wu:** resources, methodology, conceptualization, visualization. **Qihua Feng:** conceptualization, methodology, supervision, visualization, writing – review and editing, and resources.

## Ethics Statement

All procedures followed were in accordance with the ethical standards of the responsible committee on human experimentation (institutional and national) and with the Helsinki Declaration of 1964 and later versions. This study was approved by the Medical Ethics Review Committee of Maternal and Child Health Hospital of Hubei Province (Approval Number: 2024‐017‐02).

## Consent

Informed consent to be included in the study, or the equivalent, was obtained from all patients.

## Conflicts of Interest

The authors declare no conflicts of interest.

## Data Availability

The data that support the findings of this study are available from the corresponding author upon reasonable request.
